# Combined Effect of Plasma-Activated Water, Edible Coating, and Active Packaging on Cherry Tomato Shelf-Life: Kinetics and Microbiome Approach

**DOI:** 10.3390/foods15010182

**Published:** 2026-01-05

**Authors:** Syed Mudabbar Hussain Shah, Stefania Volpe, Francesca Colonna, Vincenzo Valentino, Francesca De Filippis, Elena Torrieri, Silvana Cavella

**Affiliations:** Department of Agricultural Sciences, University of Naples Federico II, 80055 Portici, NA, Italy; syedmudabbarhussain.shah@unina.it (S.M.H.S.); stefania.volpe2@unina.it (S.V.); colonnafrancesca@outlook.it (F.C.); vincenzo.valentino2@unina.it (V.V.); francesca.defilippis@unina.it (F.D.F.); cavella@unina.it (S.C.)

**Keywords:** non-thermal preservation, sodium caseinate coating, antioxidant active films, minimally processed vegetables, quality degradation kinetics, polyphenols, antioxidant capacity, metagenomic profiling, antimicrobial resistance genes

## Abstract

Cherry tomatoes are highly appreciated for their nutritional value but remain highly perishable due to rapid respiration and senescence. This study evaluated a multi-hurdle strategy combining plasma-activated water (PAW), sodium caseinate-based edible coating, and antioxidant active packaging to preserve minimally processed (MP) cherry tomatoes stored at 1 °C, 4 °C, and 8 °C for 15 days. Quality evolution was monitored through physical, chemical, nutritional, and microbiological parameters and described using pseudo-zero- and first-order kinetic models, with temperature dependence expressed by the Arrhenius equation. The combined treatment (prototype) slowed the degradation rates of pH, titratable acidity, total polyphenols, and antioxidant capacity, as reflected by consistently lower kinetic rate constants across all temperatures. Prototype samples showed better retention of polyphenols and antioxidant capacity, particularly at 1 °C and 4 °C, without detrimental effects on visual appearance. Metagenomic analysis revealed that the multi-hurdle treatment reshaped the microbial community, reducing the relative abundance of potentially problematic taxa such as *Acinetobacter johnsonii* and limiting the occurrence of antimicrobial resistance (AMR) genes at the end of storage. This study provides the first integrated assessment of PAW, edible coating, and antioxidant active packaging as a synergistic multi-hurdle strategy, demonstrating their combined ability to extend shelf life while modulating the microbiome and resistome of minimally processed cherry tomatoes.

## 1. Introduction

In recent years, consumer interest in minimally processed fruits and vegetables (MPFVs) has increased, driven by their ease of use, preservation of nutritional quality, and lower environmental footprint [1]. In 2024, the global MPFV market reached USD 377.3 billion and is projected to nearly double by 2034, reflecting increasing consumer interest in fresh-like, ready-to-eat products [2]. Cherry tomatoes (*Solanum lycopersicum* var. *cerasiforme*) represent a rapidly expanding segment within this category, appreciated for their sensory qualities and high contents of ascorbic acid, lycopene, and phenolic compounds, which contribute to antioxidant, hypoglycemic, and hypolipidemic activity [3,4]. However, despite their nutritional benefits, cherry tomatoes are highly perishable due to their elevated respiration rate, soft texture, and susceptibility to microbial spoilage, resulting in short shelf-life and significant postharvest losses [5].

To mitigate quality degradation, several mild preservation strategies have been proposed. Modified atmosphere packaging (MAP), edible coatings, and bio-based active packaging have been reported to decrease respiration, limit water loss, and preserve firmness and nutritional value of fresh-cut produce [6]. Nonetheless, the effectiveness of these technologies is often limited when used individually, particularly for high-moisture, rapidly respiring commodities. Plasma-activated water (PAW) has recently emerged as an eco-friendly sanitation alternative, generating reactive species capable of reducing microbial loads without leaving chemical residues [2,7,8]. Its efficacy has been demonstrated on strawberries [9], fennel [10], fresh-cut apples [11,12], and leafy greens [8], where PAW improved microbial safety and mitigated enzymatic and textural degradation. Edible coatings are considered a promising strategy for MPFVs, as they act as semi-permeable layers capable of regulating gas exchange, limiting water loss, and preserving firmness and nutritional quality. Likewise, active packaging systems incorporating antioxidant compounds can scavenge reactive species and limit oxidative damage during storage. Recent studies have explored the integration of coating materials with antioxidant-enriched films to enhance their preservative effect while reducing the environmental footprint of packaging.

Recent literature on cherry tomatoes has focused almost exclusively on individual mild technologies—such as PAW [13,14], coatings [15], or antioxidant packaging [16]—with only one study assessing the combined use of PAW and edible coating [10].

To date, no research has investigated how a multi-hurdle approach incorporating PAW, a protein-based edible coating and antioxidant-active packaging, influences chemical, nutritional, and microbial evolution in tomatoes under varying temperature conditions. Likewise, no available research integrates kinetic modelling with metagenomic profiling to understand both quality decay and microbiome/resistome shifts under multi-hurdle preservation.

The literature still lacks comprehensive modelling approaches capable of describing quality loss when multiple mild preservation technologies are applied simultaneously. Kinetic modelling is a powerful tool for describing the evolution of biochemical and physical attributes in fresh produce and for predicting shelf life under variable storage conditions. Pseudo-zero and pseudo-first-order reaction models, coupled with Arrhenius-type temperature dependence, have been successfully used to model changes in pH, titratable acidity, colour, firmness, and phenolic content in strawberries, tomatoes, apples, and mushrooms [17,18,19]. However, their application to MP cherry tomatoes preserved through integrated PAW–coating–active packaging systems has not yet been examined. Such models can support rapid shelf-life prediction and provide valuable information for product optimisation, cold-chain management, and industrial implementation.

We hypothesized that the synergistic combination of PAW, sodium caseinate coating, and antioxidant active packaging would slow physicochemical degradation, improve microbial stability, and extend shelf life more effectively than any single technology.

Therefore, the objectives of this study were as follows: (i) to evaluate the combined effect of PAW, a sodium caseinate–based edible coating, and antioxidant active packaging on the physical, chemical, nutritional, and microbiological quality of MP cherry tomatoes stored at 1 °C, 4 °C, and 8 °C; (ii) to model the degradation kinetics of physicochemical and nutritional parameters using zero- and first-order kinetics and to describe temperature dependence using the Arrhenius equation. (iii) to characterize the structural changes in microbial communities and antimicrobial resistance (AMR) genes during storage using metagenomic sequencing; and (iv) to determine whether the multi-hurdle prototype treatment provides superior quality retention, extended shelf life, and improved microbiological safety compared with untreated controls.

## 2. Materials and Methods

### 2.1. Materials

Sodium caseinate (bovine milk), propyl gallate, glycerol, span 80, sodium carbonate, tween 80, riboflavin, guar gum, methanol, sodium hydroxide, DPPH, and Folin–Ciocalteau reagents were purchased from Sigma-Aldrich (Milan, Italy), while the beeswax was obtained from Agraria Ughetto Apicoltura (Giaveno, Torino, Italy). All reagents were of analytical grade.

Packaging materials were provided by the Instituto Tecnológico del Embalaje (ITENE), produced as reported by Tone et al. [20]. Fresh cherry tomatoes (*Solanum lycopersicum* var. *cerasiforme*) were sourced from a supermarket located in the Portici area (Naples, Italy).

### 2.2. Methods

#### 2.2.1. Preparation of Plasma-Activated Water and Sodium-Based Coating

Plasma-activated water (PAW) was prepared following the protocol described by [21]. Briefly, plasma-processed air (PPA) was generated using a two-stage microwave-driven discharge system connected to a PLexc^®^ plasma torch (Leibniz Institute for Agricultural Engineering and Bioeconomy, Potsdam, Germany). The first stage operated at 1.3 kW and 2.45 GHz with an air flow rate of 12 slm, while the second stage operated at 1.7 kW, 2.45 GHz, and 80 slm. Plasma ignition converted compressed air into reactive nitrogen species (RNS, ~3%) and a mixture of reactive oxygen and nitrogen species (RONS) capable of oxidative microbial inactivation. The plasma-processed air stream was bubbled into 1 L of distilled water for 1 min, producing PAW enriched in long- and short-lived oxidizing species such as H_2_O_2_, NO_2_^−^, NO_3_^−^ and O_3_. The resulting PAW showed a final pH of 1.79 ± 0.01. Immediately after preparation, the PAW was frozen and shipped from Germany to Italy under controlled temperature conditions. Prior to use, PAW was thawed at room temperature (25 °C) and its pH was measured to verify consistency with the original batch. No additional activation was performed before application.

The sodium caseinate-based coating was prepared according to the optimized formulation reported by Miele et al. [22], with adaptations for this study. Briefly, sodium caseinate was dissolved in water to obtain an 8% (*w*/*v*) solution and heated at 90 °C for 1.3 h under constant stirring. Glycerol was added at a glycerol/solids ratio of 0.1 (*w*/*w*) as a plasticizer. Guar gum was incorporated at 0.2% (*w*/*v*) and stirred for an additional 30 min. Beeswax was added at 2% (*w*/*v*), together with a surfactant blend of Tween 80 and Span 80 (HLB = 9.2) at 0.25% + 0.25% (*w*/*v*). To obtain active coating, 0.13 mg mL^−1^ of propyl gallate (PG) was added after the solubilisation of SC at 20 °C [23]. The mixture was homogenized using an Ultra-Turrax at 15,000 rpm for 5 min and cooled to room temperature before application.

#### 2.2.2. Preparation of MP Cherry Tomatoes and Stored Condition

Cherry tomatoes were sorted to remove damaged fruits, rinsed, and divided into control and prototype groups. Two experimental groups were considered:

(i) Control samples: washed with warm tap water (4.5 L, 40 °C) for 2 min, drained, and dried at 30 °C and 50% relative humidity (RH) for 1 h.
(ii) Prototype samples: PAW-washed (4.5 L, 40 °C) for 2 min, coated with a sodium caseinate-based edible coating, drained and dried as control samples. Dried cherry tomatoes were coated using an active edible coating based on sodium caseinate, guar gum, beeswax, and propyl gallate. Samples were dipped into the edible coating for 2 min and then quickly withdrawn, drained for 2 min, and dried for 1 h at 30 °C and 60% relative humidity.

Control samples were packed in PP 20 μm/PP 50 μm pouches, whereas prototype samples were packed in antioxidant PP 20 μm/PP + 10% orange peel extract 50 μm films. Barrier properties are reported by Khan et al. [16]. Packages contained 150–200 g of fruit and were stored at 1 °C, 4 °C, and 8 °C for 15 days. Sampling was performed before storage and after 2, 5, 7, 9 and 15 days.

#### 2.2.3. Experimental Design

The experimental design was structured as a full factorial arrangement to evaluate the effects of three independent variables: processing method (2 levels), storage time (6 levels), and storage temperature (3 levels) on the quality attributes of tomato samples. The experimental plan resulted in 36 treatment combinations (2 × 6 × 3), each performed in triplicate. The response variables included colour parameters, pH, titratable acidity, antioxidant capacity, total phenolic content, total aerobic microbial count, yeasts and moulds, and *Enterobacteriaceae*.

#### 2.2.4. Appearance and Colour Evaluation

The visual quality of minimally processed cherry tomatoes was assessed by acquiring images at each storage interval, both in packaged and unpackaged conditions, using a lightbox system (LED illumination, light intensity 4500 lux, colour temperature 5000–5500 K). Colour measurements were carried out with an electronic eye (VA400 IRIS, Alpha MOS, Toulouse, France) equipped with a CCD camera (2592 × 1944 pixel resolution, 24-bit depth) [23]. The acquired images were initially processed in the RGB colour space and subsequently converted to the CIE L*, a*, b* colour coordinates using Alphasoft software (version 16.0). The L*, a*, and b* values were determined at multiple locations on the tomato surface, and the overall colour variation was expressed as total colour change (TCC) [24].

#### 2.2.5. Physicochemical Analysis

MP cherry tomatoes were homogenized using a blender and then filtered. The pH of the resulting tomato juice was measured using a digital pH meter (DPH-2, Atago Co., Tokyo, Japan), with three measurements taken for each sample. Titratable acidity (TA) was determined by weighing 5 g of the homogenized tomato solution into a flask and titrating with 0.1 M NaOH until a pH of 8 was reached. Results are expressed as millilitres of titratable acidity. All assays were performed in triplicate for each treatment. Values at different storage times were normalised to the values of the samples at time 0, where the initial pH and TA were 4.12 ± 0.01 and 3.52 ± 0.01, respectively.

#### 2.2.6. Determination of Total Polyphenol Content (TPC)

The total phenolic content (TPC) was determined as described by Di Giuseppe et al. [25]. A total of 1 g of the homogenized sample was diluted with 10 mL of sodium carbonate (6%). The solution was filtered with a bench paper. 0.5 mL of filtrate was mixed with 2.5 mL of Folin–Ciocalteau reagent (10%) and 2 mL of sodium bicarbonate (6%). The sample was incubated in the dark for 1 h at 35 °C, then for 1 h at 6 °C. The absorbance of the incubated sample was measured at 760 nm against a blank (2.5 mL of Folin–Ciocalteau reagent and 2.5 mL of sodium bicarbonate), using a UV–VIS spectrophotometer UV-550 (Jasco, Tokyo, Japan). The TPC was expressed as mg gallic acid equivalent per g of dry weight (mg GAE eq g^−1^_dw_), using a calibration curve in the range 0–8 mg mL^−1^. The results, at each storage time, were normalised to the initial TPC value (1.9 ± 0.05 mg GAE eq g^−1^_dw_).

#### 2.2.7. Determination of Antioxidant Capacity

A 0.5 g aliquot of the homogenized sample was extracted with 20 mL of pure methanol in an ultrasonic bath for 30 min, followed by centrifugation at 10,000 rpm for 10 min at 4 °C. Then, 100 μL of the filtered supernatant was mixed with 3.9 mL of 0.1 mM DPPH solution and incubated in the dark at room temperature for 30 min. Absorbance was measured at 517 nm using a UV–VIS spectrophotometer (UV-550, JASCO, Tokyo, Japan) against methanol as a blank.

Antioxidant capacity (AOC) was expressed as mg Trolox equivalents per g of dry weight (mg Trolox g^−1^_dw_) using a Trolox calibration curve (0–625 mg mL^−1^). Values at each storage time were normalised to the initial AOC (16.85 ± 2.05 mg Trolox g^−1^_dw_).

#### 2.2.8. Microbiological and Metagenomic Analyses

Samples were serially decimal diluted and aliquoted in plates of suitable media for each microbial group: plates of Plate Count Agar (PCA) for the enumeration of mesophilic bacteria incubated at 30 °C for 3 days; plates of Violet Red Bile Glucose agar (VRBGA) incubated at 37 °C for 48 h to count *Enterobacteriaceae*; and plates of Dichloran Rose-Bengal Chloramphenicol agar (DRBC) incubated at 28 °C for 4 days to count yeasts and moulds.

For metagenomics analysis, the microbial DNA was extracted from the prototype and control samples. DNA extraction from samples stored at 1 °C gave low yields. Therefore, metagenomics was carried out only on samples stored at 4 °C and 8 °C. All the samples were analysed in triplicate. Briefly, samples were weighted and suspended in a 5:1 ratio in Phosphate Buffered Saline (PBS). Microorganisms were detached from the surface of tomatoes without breaking the matrix to avoid the release of plant cells. One hundred mL of supernatant was centrifuged at 6500× *g* for 15 min in order to precipitate the microbial cells. DNA extraction from the biomass was performed as previously described [26]. DNA concentration was estimated using the Qubit dsDNA HS assay kit (Thermo Fisher Scientific, Waltham, MA, USA), then Whole Metagenome Shotgun Sequencing was performed with an Illumina HiSeq 4000 instrument (Illumina, San Diego, CA, USA), following the manufacturer’s procedure, leading to 2 × 150 bp raw reads.

The species-level profiles were estimated through MetaPhlAn (version 4.0.6; 10.1038/s41587-023-01688-w) using default settings. Raw reads were assembled into contigs using MegaHIT (version 1.3.1, 10.1093/bioinformatics/btv033) with default options, and antimicrobial resistance (AMR) genes were predicted from contigs through abricate (version 1.0.1; https://github.com/tseemann/abricate accessed on 1 April 2023) relying on the CARD (10.1093/nar/gkw1004), Resfinder (10.1093/jac/dks261) and ARG-ANNOT (10.1128/AAC.01310-13) databases, filtering out all the alignments with percent identity and coverage below 80%. Read per Kilobase per Million Mapped Reads (RPKM) abundance of each AMR gene was calculated as previously described by Mortazavi et al. [27].

#### 2.2.9. Kinetic Modelling

Changes in pH, TA, TPC, and AOC were modelled using pseudo-zero (Equation (1)) and first-order kinetics (Equation (2)) [28]:

(1) $$\left[A\right]={\left[A\right]}_{0}-kt$$

(2) $$ln\left[A\right]=ln{\left[A\right]}_{0}-kt$$

where $A_{0}$ and A represent the initial and actual values of the quality parameters, k is the quality parameter decay rate constant [day^−1^], and *t* is the time [day].

The Arrhenius model was used to describe the temperature-dependent decay rate constant:

(3) $${lnk}_{ref}=\left(\frac{-E_{a}}{R}\right)\cdot \left(\frac{1}{T_{ref}}\right)+{lnA}_{0}$$

where *k_ref_* is the reaction rate at reference temperature (*T_ref_* = 277.45K), *A*_0_ is the frequency factor (or pre-exponential factor), *Ea* is the activation energy, and *R* is the universal gas constant (8.314 J mol^−1^ K^−1^); *T* is the temperature (K).

#### 2.2.10. Statistical Analysis

All experiments were performed in triplicate using three independent batches of fresh cherry tomatoes. Results are reported as mean ± standard deviation. Prior to statistical analysis, data were tested for normality using the Shapiro–Wilk test and for homogeneity of variances using Levene’s test. When the assumptions were met, a multifactorial analysis of variance (ANOVA) was applied to evaluate the main effects of processing method, storage time, and storage temperature, as well as their two- and three-way interactions, on the measured quality parameters. Mean comparisons were performed using Duncan’s multiple range test at a significance level of *p* < 0.05. When appropriate, pairwise comparisons between the two processing methods at fixed storage time and temperature were additionally performed using independent-sample *t*-tests. All statistical analyses were carried out using SPSS software (version 25.0; SPSS Inc., Chicago, IL, USA).

Kinetic parameters were estimated by regression analysis using Microsoft Excel (version 2409). Zero- and first-order kinetic models were selected based on the coefficient of determination (*R*^2^), root mean square error (*RMSE*), and the absence of systematic patterns in residuals. The temperature dependence of the rate constants was described using the Arrhenius equation.

Metagenomic data analysis was conducted in R software (version 4.4.1). Species-level bar plots were generated using the ggplot2 package, while hierarchical clustering of antimicrobial resistance (AMR) gene families was performed using the pheatmap package. Spearman’s rank correlation coefficient (ρ) was calculated to assess correlations between the abundance of microbial taxa and the RPKM abundance of AMR gene families using the corr.test function from the psych package. Significant correlations (*p* < 0.05) were visualized using the corrplot package.

## 3. Results

### 3.1. Appearance and Colour Parameters

**Figure 1:**
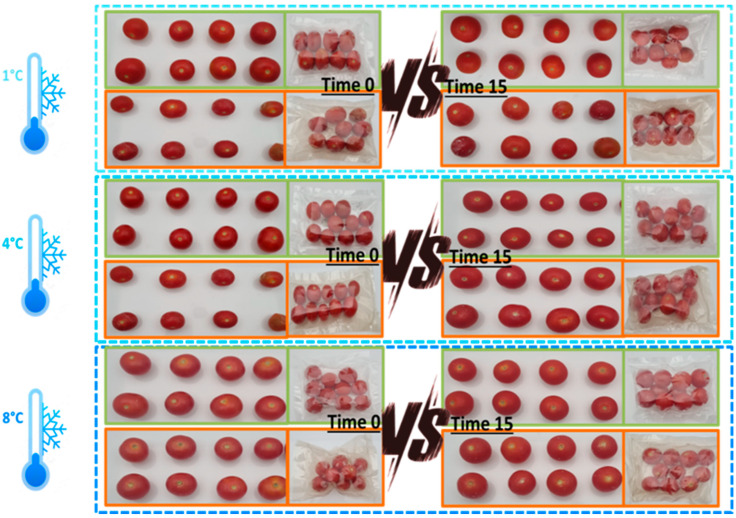
Changes in appearance in control and prototype groups of cherry tomatoes during storage at different temperatures. Images of cherry tomatoes (control (green line)—prototype (red line)) stored at 1 °C, 4 °C and 8 °C for 15 days.

As shown in Figure 1, at all temperatures, the treatments did not affect the visual appearance of MP cherry tomatoes up to 15 days of storage. Both control and prototype groups maintained similar levels of colour intensity and uniformity, with no notable surface defects or wrinkling observed over time. These results indicate that the implemented technologies did not compromise the visual appearance of the MP cherry tomatoes.

Colour plays a key role in influencing consumer decisions, attracting attention, shaping preferences, and increasing the appeal and acceptance of fruits and vegetables [29,30]. The colour parameters of control and prototype groups, at each storage temperature and time, are shown in Table 1. ANOVA shows that the time has a significant effect on L*, a*, b*, and Δ*E* (*p* < 0.05), whereas the treatment and the temperature had a significant impact on the colour parameters a*, b*, and Δ*E* (*p* < 0.05 to *p* < 0.001). The interaction effect between time and temperature was statistically significant for all the parameters (*p* < 0.001). For all samples and at all temperatures, the L* value decreased from the initial value of 42 ± 2 to an average value of 36 ± 2 after two days of storage and then remained constant until 15 days of storage. At 1 °C, a* value for all samples increased from 14 ± 3 to 20 ± 3 after 9 days but then decreased again to 14 ± 3 by day 15. The same trend was observed for b*, whose value increased from 14 ± 3 to 19 ± 2 by day 2, then remained constant for up to 9 days and again decreased to 14 ± 2 on day 15.

Thus, with a slight variation over time, it is not possible to highlight a clear trend of a* and b* as a function of time. At 4 °C and 8 °C, a* value increased after two days up to a value of around 20 ± 1, then remained constant for up to 15 days. A similar trend was observed for b*, whose value increased during time up to 20 ± 1, with significant differences since day 2. For the a* parameter, although the significant effect of temperature, the results did not highlight a clear dependence (the value of a* at 1 °C and 8 °C were not statistically different, whereas, at 4 °C, it assumed an average higher value). At all the temperatures studied and for all samples, the Δ*E* increased from 0 to around 10 after 15 days of storage, with a significant difference since day 2.

In a previous work, a significant effect of PAW and casein-based coating on the colour changes of cherry tomatoes was observed, with a preservative impact over time [7]. Earlier findings reported that the different types of edible coating can delay the total colour changes in MPFV by minimizing the oxidation rate and ethylene production [31,32]. Thus, the different results obtained in this work could highlight a negative role of the propyl gallate added to the coating and of the active packaging on the colour parameters. Due to the slight changes in the colorimetric parameters, they were not considered as critical quality indices for the product’s shelf-life.

In addition, colour was excluded from kinetic modelling because the calculated slopes of L*, a* and b* values over storage time were not statistically different from zero (*p* > 0.05), and R^2^ values obtained from preliminary fits were below 0.30. These quantitative indicators confirm the absence of a meaningful monotonic trend, making kinetic modelling unreliable and biologically unjustified. The stability of colour parameters therefore represents an outcome of treatment efficacy rather than a limitation of the modelling approach. In kinetic studies, modelling is justified when a parameter exhibits a clear monotonic trend, allowing reliable estimation of rate constants. As reported by Yi et al. [18], colour parameters that do not display directional or progressive changes cannot be fitted accurately to kinetic models. In our case, the absence of meaningful variation in colour produced near-zero slopes that were not statistically distinguishable from noise, preventing reliable kinetic modelling.

### 3.2. Physicochemical Properties of Cherry Tomatoes

**Figure 2:**
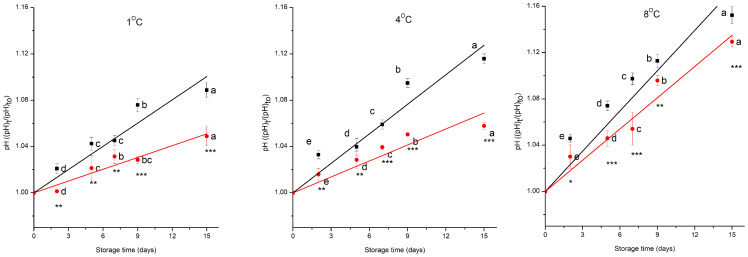
Changes in normalised pH, control (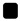) and prototype (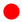) groups, of MP cherry tomatoes during storage at different temperatures. Values represent the mean ± SD of three replicates. Different letters indicate significant differences over time (*p* < 0.05). Asterisks denote treatment effects: * *p* < 0.05, ** *p* < 0.01, *** *p* < 0.001. The lines **—** and **—** are the data predicted by the model for control and prototype groups, respectively.

As a general trend, the pH of cherry tomatoes changes during storage, and a lower pH is associated with a slower respiration rate, meaning improved preservation condition [33]. Moreover, the pH of a fruit sample affects its taste and flavour during postharvest storage [34]. ANOVA showed that all the independent variables (treatment, time, and temperature) had a significant effect on the pH of the cherry tomatoes (*p* < 0.001), the interaction effects among factors were also significant(*p* < 0.001). The initial pH of the cherry tomatoes was about 4.12 ± 0.01; during storage, the pH of the control group increased by 8%, 11%, and 13% whereas that of the prototype group increased by 5%, 6%, and 11%, respectively, at 1 °C, 4 °C, and 8 °C. Figure 2 shows the normalised pH data respect to the value of control sample at time zero as a function of storage time, for both the control and prototype groups, at 1 °C, 4 °C, and 8 °C.

These results indicate that microbial and enzymatic activities, which increase with temperature, lead to greater pH changes [35]. Furthermore, the combination of PAW, edible coating, and antioxidant packaging effectively mitigates the metabolic process, altering the pH during storage. Similar results have been reported for cherry tomatoes preserved with a chitosan nano-biopolymer/citrus paradisi peel oil system [36] and with a pectin edible coating [37]. Both studies demonstrated that the pH of coated tomato samples increases less intensively than the control ones, the coating having the effect of slowing down the rate of respiration and thus the consumption of organic acids.

The change of pH during storage time follows a pseudo-zero-order kinetic model (Figure 2, Table 2). The goodness of the model is demonstrated by the statistical data (R^2^, *RMSE*) (Table 2). The low *RMSE* values confirm the accuracy of the predictions. When interpreted relative to the magnitude of the measured variables, the *RMSE* corresponds to a very small relative error, in several cases comparable to or lower than the experimental uncertainty. This indicates that the discrepancies between predicted and observed values are not only statistically small but also negligible from a practical standpoint. The observed behaviour is physiologically justified by the metabolic processes that occur in MP cherry tomatoes. Organic acids are gradually consumed through respiration and decarboxylation reactions, producing CO_2_ and water, and these pathways progress at a relatively constant rate under steady temperature conditions [35]. Because the consumption of acids is regulated primarily by enzymatic respiration activity rather than by the absolute concentration of acids at any given moment, the pattern aligns with zero-order dependence [38]. The combination of the preservative technologies implemented significantly (*p* < 0.001) reduces the kinetic constant of pH change by 50% at 1 °C, by 56% at 4 °C, and by 18% at 8 °C, compared to the control group.

The Arrhenius equation well predicted the effect of temperature on the kinetic constants (*R*^2^ > 0.97) of both groups. As given in Table 3, the treatment has a significant impact on the activation energy [19] (*p* < 0.001). The Ea for the control and prototype groups was 51 ± 2 kJ mol^−1^ and 93 ± 3 kJ mol^−1^, respectively, highlighting that the prototype group was more sensitive to temperature variation than the control group during storage. Our findings were aligned with previous studies where the pseudo-zero-order kinetic model effectively described changes in pH of cherry tomatoes treated with high-voltage electrostatic fields [39]. Moreover, the change in pH of strawberries stored at different temperatures was also described with a pseudo-zero-order kinetic model [17].

**Figure 3:**
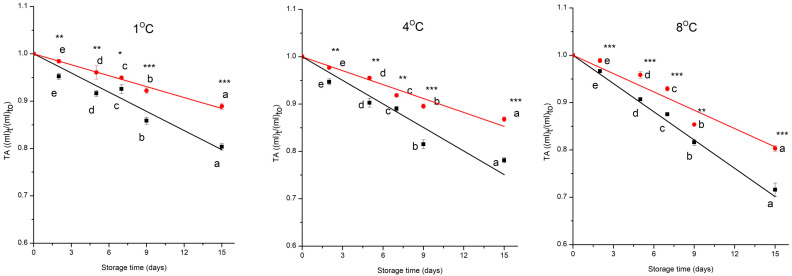
Changes in normalised titratable acidity, control (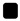) and prototype (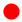) groups, of MP cherry tomatoes during storage at different temperatures. Values represent the mean ± SD of three replicates. Different letters indicate significant differences over time (*p* < 0.05). Asterisks denote treatment effects: * *p* < 0.05, ** *p* < 0.01, *** *p* < 0.001. **—** and **—** are the data predicted by the model for control and prototype groups, respectively.

Titratable acidity (TA) plays an important role in determining fruit flavour, taste, and sensory qualities [18,40]. The normalised value of TA during storage, for both groups of MP cherry tomatoes at all the temperatures, is reported in Figure 3. ANOVA showed that the time, the treatment, and the storage temperature have a significant effect on TA (*p* < 0.001). The interaction effect was also significant, a part of the interaction between temperature and treatment. At 1 °C, 4 °C, and 8 °C, the control group showed a decrease of 20%, 22%, 28%. In contrast, the prototype group exhibited a reduction of 11%, 13% and 20% over the 15 days, respectively. TA values steadily dropped during storage because of decreasing organic acid contents, primarily citric acid, in contrast to pH, which rose over the postharvest storage period [41]. The prototype group showed a significantly higher TA than the control one (Figure 3), indicating that combined preservative technologies, mainly the coating and the antioxidant packaging, effectively slowed down metabolic activities and organic acid oxidation during postharvest [42].

A similar response of the TA was reported for the guava [43], tomatoes [44], and cherry tomatoes [45] treated with gum arabic, coating of bitter almond gum-fish gelatin conjugates, and chitosan/casein containing Origanum vulgare L. essential oil, respectively.

TA changed according to a pseudo-zero-order kinetic model (Figure 3, Table 2). The combination of high R^2^, low *RMSE*, and the lack of systematic residual patterns supports the robustness of the proposed models within the studied domain. The observed behaviour is consistent with the metabolic profile of fresh-cut tomatoes, in which organic acids such as citric and malic acids serve as substrates in the tricarboxylic acid (TCA) cycle during respiration. The prototype group had a lower kinetic constant value than the control group; the reduction was of 38%, at 1 and 4 °C, and of 32%, at 8 °C, compared to the control group. As the temperatures increased from 1 °C to 8 °C, the k-values significantly (*p* < 0.05) increased for both groups.

The effect of the temperature on the kinetic constant has been predicted using the Arrhenius equation. As reported in Table 3, the *Ea* for the control group was 35 ± 3 kJ mol^−1^, which was significantly (*p* < 0.05) lower than the prototype group (46 ± 1 kJ mol^−1^). The results are consistent with the previous studies, where the kinetics of TA of different MPFV, such as strawberries, Shiitake mushrooms, and Kiwifruit, were also well described by the pseudo-zero-order kinetic model [17,18,19].

### 3.3. Changes in Total Polyphenol Content During Storage

**Figure 4:**
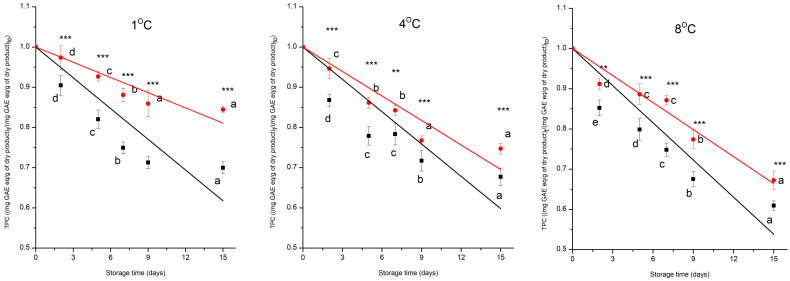
Changes in normalised total polyphenol content (TPC), control (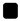) and prototype (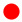) groups, of MP cherry tomatoes during storage at different temperatures. §Values represent the mean ± SD of three replicates. Different letters indicate significant differences over time (*p* < 0.05). Asterisks denote treatment effects: ** *p* < 0.01, *** *p* < 0.001.). **—** and **—** are the data predicted by the model for control and prototype groups, respectively.

The normalised values of TPC as a function of the storage time, at different temperatures, are illustrated in Figure 4. The TPC showed a significant decreasing trend in both control and prototype groups, achieving the control group a reduction of 30%, 32% and 39% and the prototype group a reduction of 16%, 25% and 33% over 15 days at 1 °C, 4 °C, and 8 °C, respectively. ANOVA showed that all the independent variables (time, treatment, and temperature) had a significant effect on the TPC of the MP cherry tomatoes (*p* < 0.001), significant was also the interaction effect among factors (*p* < 0.001). Combined preservative technologies were evidently successful in slowing down the enzymatic and chemical degradation of the polyphenols. It was already proven that treatment with PAW, alone or combined with the use of antimicrobial proteins, prevents damage to the cell structure of cherry tomatoes, thus delaying the degradation of polyphenols [46]. The results of this research are consistent with earlier studies by [47], which discussed the preservation of polyphenol content in tomatoes using coatings.

The TPC degradation during storage followed a pseudo-zero-order kinetic model (Figure 4, Table 2). The high *R*^2^ and low *RMSE* values confirm the accuracy of the predictions. In addition, the residual analysis does not reveal evident systematic deviations across the investigated range. Residuals are evenly distributed around zero, with no clear trends of overestimation or underestimation at low or high values, suggesting the absence of significant bias in the model predictions Minor local deviations can be attributed to experimental noise or simplifications inherent to the modelling assumptions rather than to structural model inadequacies.

This behaviour is characteristic of oxidative and enzymatic reactions involving polyphenols in fresh-cut produce. Polyphenols are substrates for polyphenol oxidase (PPO) and peroxidase (POD), enzymes that catalyse their conversion into quinones in the presence of oxygen. Because the reaction rate depends directly on substrate availability, phenolic degradation typically follows first-order dependence. In addition, reactive oxygen species (ROS) generated during storage promote non-enzymatic oxidation, further contributing to concentration-dependent losses [48]. The kinetic constant of the prototype group, compared to the control one, was reduced by 40%, 30%, and 26%, over 15 days at 1 °C, 4 °C, and 8 °C, respectively (*p* < 0.01). The k-values of TPC were significantly lower at 1 °C compared to samples stored at 4 °C and 8 °C (*p* < 0.05).

To assess the effect of temperature, Arrhenius equation parameters have been estimated. As shown in Table 3, the treatment had a significant impact on the *Ea* (*p* < 0.05). The control group had a lower *Ea* value (19 ± 3 kJ mol^−1^) than the prototype group (38 ± 6 kJ mol^−1^), pointing out that when the combined technologies were used, the energy barrier of polyphenol degradation was higher, but the rate changed more quickly with temperature. Similar findings for the total polyphenol content of tomatoes are reported in the literature; the combined effects of ultraviolet-C irradiation and ultrasound on TPC kinetics were studied. The TPC was best described by a first-order kinetic model, with the treated group showing activation energy (*Ea* = 135 kJ mol^−1^), indicating the sensitivity of TPC to temperature changes. Muley et al. [30] investigated the impact of storage temperature (5 °C, 10 °C, 20 °C, 26 °C, and 32 °C) on freshly harvested strawberries and assessed the physical and biochemical changes during storage, and found that the TPC data were also best fitted with the pseudo-zero-order kinetic model with *Ea* = 31 kJ mol^−1^.

### 3.4. Changes in Antioxidant Capacity During Storage

**Figure 5:**
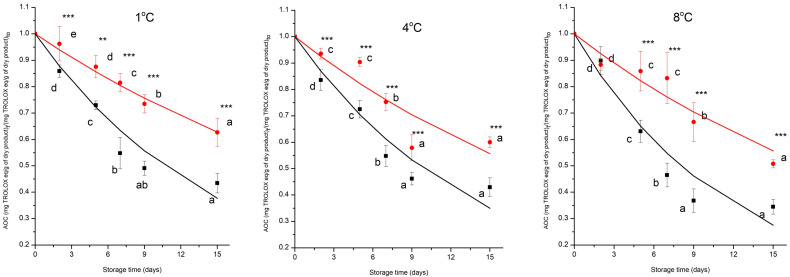
Changes in normalised antioxidant capacity (AOC) in control (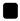) and prototype (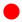) groups of MP cherry tomatoes during storage at different temperatures. Values represent the mean ± SD of three replicates. Different letters indicate significant differences over time (*p* < 0.05). Asterisks denote treatment effects: ** *p* < 0.01, *** *p* < 0.001. **—** and **—** are the data predicted by the model for control and prototype groups, respectively.

The trend of antioxidant capacity of MP cherry tomatoes is illustrated in Figure 5. The normalised values of AOC of the control and prototype groups decreased significantly (*p* < 0.05) from day 0 to day 15 of the storage period at all temperatures investigated. The control group presented a drop of 10%, 12%, and 15%, while the prototype a reduction of 8%, 10%, and 13%, from day 0 to day 15 at 1 °C, 4 °C, and 8 °C, respectively, underwent a substantial depletion during storage time from day 0 to day 15, retaining 18% of antioxidant capacity due to combined technology. ANOVA showed that all the independent variables (time, treatment and temperature) had a significant effect on the AOC of the MP cherry tomatoes (*p* < 0.001) and a significant interaction effect among factors has been found (*p* < 0.001).

The general trend indicated that the prototype group consistently maintains a higher antioxidant capacity compared to the control group across all temperatures. These results can be attributed to the combined technologies, providing protective effects against oxidative degradation, microbial activity, and enzymatic breakdown, thereby slowing the decline in antioxidant capacity over time. Higher storage temperatures (8 °C) are associated with a faster decrease in antioxidant capacity, likely due to increased rates of oxidative and enzymatic degradation. According to the literature on the impact of coatings on tomato fruit’s antioxidant capacity, the application of coatings slows down the process of the fruit’s antioxidant capacity being reduced throughout the post-harvest period. It also shows that fresh-cut tomatoes stored at 10 °C exhibited higher antioxidants than those stored at 15 °C and 20 °C.

Antioxidant capacity decreased during storage time for all samples following a pseudo-first-order kinetic model, with the rate constant (*k*) increasing with temperature for both groups (Figure 5, Table 2). The goodness of fit achieved by the model is reflected in the high coefficients of determination and the low prediction errors. This indicates that the experimental antioxidant data are well reproduced throughout the analysed range. Examination of the residuals shows no evidence of systematic bias, as deviations are randomly scattered around zero with no preferential trends at low or high antioxidant values The small residual discrepancies observed can be ascribed to the inherent experimental uncertainty of antioxidant assays and to necessary model simplifications, rather than to deficiencies in the model formulation itself.

In cherry tomatoes, antioxidant capacity is largely driven by polyphenols, ascorbic acid and other redox-active metabolites whose degradation involves concentration-dependent oxidative and enzymatic pathways. As these compounds react with reactive oxygen species (ROS) or serve as substrates for PPO and POD, the reaction rate decreases progressively as the antioxidant pool becomes depleted, consistent with first-order behaviour [42]. Furthermore, the k-values of AOC were significantly (*p* < 0.001) different for both control and prototype groups, and the k-values of the prototype groups were lower than the control groups across all the temperatures (Table 2). The k-values of the prototype group, compared to the control group, decreased by 52%, 44% and 39%, respectively, at 1 °C, 4 °C, and 8 °C, with a positive effect on the preservation of the AOC.

The dependence of the k-value by temperature was described by the Arrhenius equation; the treatment had a highly significant effect on the *Ea* (*p* < 0.001), with the control group having a lower *Ea* (24 ± 2 kJ mol^−1^) than the prototype group (46 ± 1 kJ mol^−1^). The results of our study align with previous findings, demonstrating the effectiveness of active coatings in preserving antioxidant capacity and following a pseudo-first-order kinetic model for fennel at different storage temperatures, and the *Ea* of the antioxidant loss was reduced by using a coating preservation treatment [15].

### 3.5. Microbiological and Metagenomic Results

**Figure 6:**
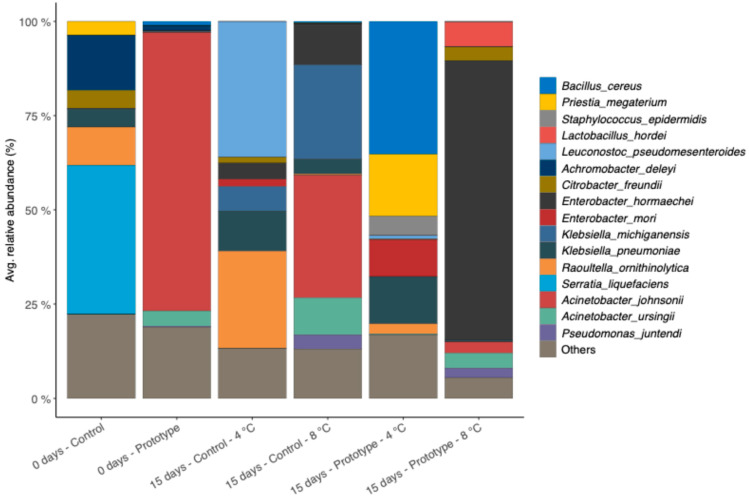
Bar chart showing the species-level composition of Prototype and Control samples at baseline and after 15 days of storage at 4 °C and 8 °C. Only species with an average relative abundance > 2% are shown.

Microbial counts of control and prototype groups during storage at 1 °C, 4 °C, and 8 °C up to 15 days are shown in Table 4a–c. No differences in the total aerobic microbial loads were found between the control and prototype groups at the end of storage at all storage temperatures. However, values at 4 and 1 °C were significantly lower than those at 8 °C (*p* < 0.05). In samples stored at 8 °C, we observed lower *Enterobacteriaceae* loads in the prototype vs control group from 9 days of storage onwards, suggesting that the treatment might effectively limit the overgrowth of this taxonomic group during the shelf-life of tomatoes. On the contrary, yeasts/moulds were higher in prototype groups stored at 4 °C and 8 °C, while they were always below the detection limit at 1 °C. Interestingly, PAW treatment led to a change in the microbiome taxonomic composition at baseline, with a decrease in the abundance of several *Enterobacteriaceae* species (*Serratia liquefaciens*, *Raoultella ornithinolytica*), while *Lactobacillus* spp. prevailed in the prototype (Figure 6). The difference observed at baseline was reflected in different microbial dynamics during the storage (Figure 6). While PAW seems to be effective in reducing several *Enterobacteriaceae* species, it probably had a different effect of different species, leading to the dominance of other members of the microbial community. Indeed, complex interactions within the different species (e.g., cross-feeding, antagonism) may lead to different results in microbiome assembly. In particular, we highlighted a drop in the abundance of *Acinetobacter johnsonii*, which shifted from an average of 73.9 ± 3.6% at baseline to 2.97 ± 2.75% after 15 days of storage at 8 °C. This species has been frequently isolated from fruits and vegetables [49]. *A johnsonii* is commonly found in food processing environments, including vegetable producing facilities [50,51] from where it can be easily spread to the products. It has been recently advised as a reservoir of AMR genes potentially acquired through horizontal gene transfer events [50] and a recent wide metagenomics survey of food processing resistome highlighted it as one of the most important contributors to AMR in food industry (particularly β-lactams), even more than the well-known ESKAPEE pathogens [51]. Therefore, although specifically designed studies would be necessary to validate these results, PAW application seems effective in reducing *A. johnsonii* proliferation, improving raw vegetable safety.

**Figure 7:**
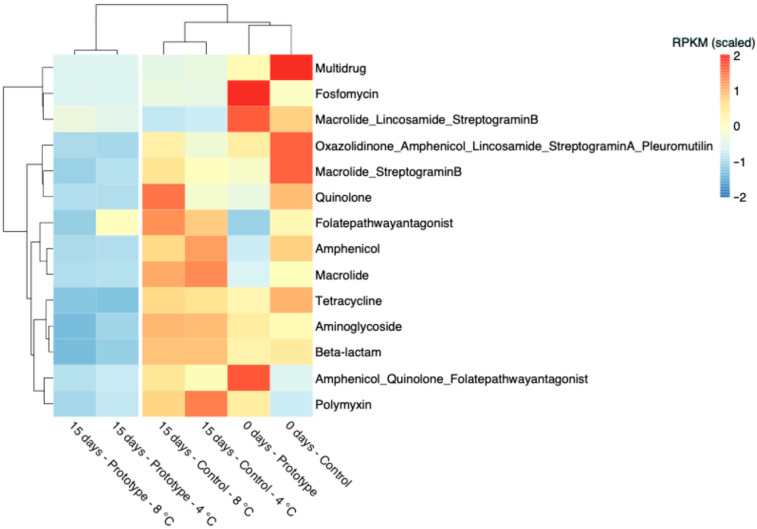
Ward’s linkage clustering based on the Canberra distance of the scaled Reads per Kilobase per Million Mapped Reads (RPKM) abundance of antimicrobial resistance gene families.

Furthermore, after 15 days of storage (Figure 7), a drop in the abundance of genes involved in resistance to different antibiotic classes was observed in prototype samples compared with control samples, suggesting that the treatment might limit the proliferation of bacterial species encoding genes linked with resistance to antimicrobial compounds, regardless of the storage temperature (Figure 7).

**Figure 8:**
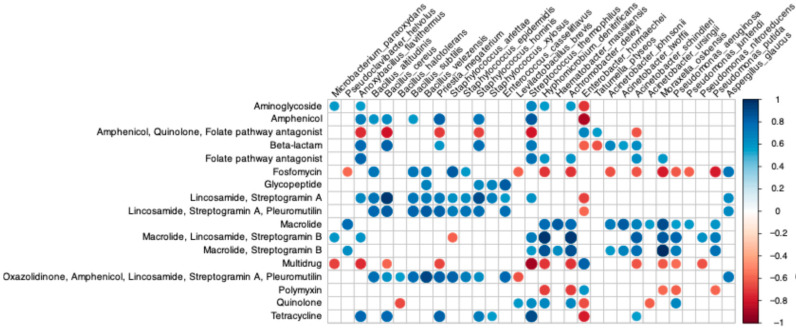
Correlogram showing the Spearman’s ρ rank correlation coefficient between RPKM of AMR gene families (on rows) and relative abundance of microbial taxa showing an average relative abundance > 0.5 (on columns). Blue values denote positive correlations. Only significant correlations (*p*-value < 0.05) are shown.

Microbial taxa potentially harbouring these AMR gene families included several *Acinetobacter* species (e.g., *A. johnsonii*, *A. lwoffii*, *A. schindleri*), that correlated positively with resistance to macrolide, β-lactams and streptogramin B, as well as *Bacillus* spp. (including *B. cereus*), showing positive associations with genes encoding for resistance to lincosamide, streptogramin A and pleuromutilin (Figure 8).

## 4. Conclusions

This study investigated the impact of a multi-hurdle preservation strategy, plasma-activated water (PAW), edible coating, and active antioxidant packaging, on the appearance, pH value, titratable acidity, colour, total polyphenol content, antioxidant capacity, and microbiological properties of MP cherry tomatoes stored at 1 °C, 4 °C, and 8 °C. The combined treatment improved appearance, pH stability, and TA retention, enhanced antioxidant capacity, and slowed the degradation of polyphenols, although slight colour changes were observed compared with the control. The contributions of each hurdle were distinct: PAW primarily reduced surface microbial load and initial oxidative stress; the edible coating limited oxygen transfer and moisture loss, helping maintain metabolic stability; and the active packaging further protected bioactive compounds by scavenging reactive oxygen species. Together, these mechanisms resulted in slower deterioration rates, well described by zero and first-order kinetic modelling, with temperature dependence well described by the Arrhenius equation. The combined treatment also led to a change in the microbiome taxonomic composition, particularly limiting the proliferation of bacterial species linked to antimicrobial resistance, regardless of the storage temperature.

Overall, the physical, chemical, nutritional, and microbiological properties of minimally processed cherry tomatoes were influenced by both the treatments and storage temperatures. Lower temperatures (1 °C and 4 °C) helped to better preserve the quality of MP cherry tomatoes. Our findings also demonstrated the feasibility of predicting the shelf-life and quality changes of MP cherry tomatoes at different storage temperatures using pseudo-zero and first-order kinetics in combination with the Arrhenius equation. Moreover, these findings would help consumers to determine the edible time for MP cherry tomatoes and the distributors to think about stock and sales strategies. A limitation of this study is the inability to isolate the individual effects of these technologies as they were intentionally applied as an integrated multi-hurdle system. However, the individual effect was already investigated and known as discussed in the Introduction section.

Despite promising performance of this study, the industrial application of the proposed multi-hurdle system presents certain limitations. PAW generation requires dedicated equipment and energy input, while edible coating application may increase processing time and operational complexity. Additionally, active packaging materials may raise production costs and require regulatory approval depending on formulation. These factors must be balanced against shelf-life extension benefits, waste reduction and improved safety. Further techno-economic and life-cycle assessments are therefore necessary before large-scale adoption.

These technologies collectively support sustainability goals by extending shelf-life, reducing waste and minimising chemical sanitiser use. Future work should evaluate process optimisation, cost–benefit implications and applicability to other fruits and vegetables under diverse storage conditions.

## Figures and Tables

**Table 1:** Colour parameters (L*, a*, b*, and Δ*E*) of control and prototype groups of MP cherry tomatoes, stored at different temperatures up to 15 days.

		Temperature	Parameters	Storage Days					
				**0**	**2**	**5**	**7**	**9**	**15**
Control Group		1 °C	L*	42 ± 2 ^d^	34 ± 3 ^a^	38 ± 2 ^c^	35 ± 2 ^a^	37 ± 2 ^b^	39 ± 2 ^c^
			a*	14 ± 3 ^a^	18 ± 2 ^b^	18 ± 3 ^bc^	20 ± 4 ^c^	20 ± 2 ^c^	13 ± 3 ^a^
			b*	15 ± 3 ^a^	20 ± 3 ^d^	18 ± 4 ^b^	20 ± 4 ^cd^	18 ± 3 ^bc^	14 ± 2 ^a^
			Δ*E*	0	9 ± 3 ^cd^	7 ± 4 ^b^	10 ± 4 ^d^	8 ± 3 ^bc^	5 ± 2 ^a^
		4 °C	L*	40 ± 2 ^b^	36 ± 2 ^a^	36 ± 2 ^a^	35 ± 2 ^a^	36 ± 2 ^a^	36 ± 4 ^a^
			a*	16 ± 2 ^a^	20 ± 3 ^b^	20 ± 3 ^b^	21 ± 3 ^bc^	20 ± 4 ^bc^	22 ± 3 ^c^
			b*	15 ± 2 ^a^	19 ± 3 ^cd^	19 ± 3 ^c^	21 ± 3 ^d^	17 ± 3 ^b^	21 ± 3 ^d^
			Δ*E*	0	8 ± 4 ^a^	8 ± 3 ^a^	10 ± 3 ^b^	8 ± 3 ^a^	10 ± 4 ^b^
		8 °C	L*	42 ± 1 ^d^	38 ± 2 ^c^	37 ± 2 ^bc^	35 ± 2 ^a^	35 ± 3 ^a^	37 ± 2 ^b^
			a*	16 ± 2 ^a^	18 ± 2 ^b^	18 ± 3 ^b^	20 ± 2 ^b^	19 ± 3 ^b^	18 ± 3 ^b^
			b*	15 ± 2 ^a^	17 ± 2 ^b^	15 ± 3 ^a^	19 ± 3 ^c^	18 ± 3 ^cd^	17 ± 3 ^bc^
			Δ*E*	0	5 ± 3 ^a^	6 ± 2 ^ab^	10 ± 3 ^d^	8 ± 4 ^b^	6 ± 3 ^bc^
Prototype Group		1 °C	L*	41 ± 2 ^d^	36 ± 3 ^b^ *	34 ± 3 ^a^***	35 ± 3 ^ab^ *	35 ± 2 ^ab^	38 ± 4 ^c^
			a*	14 ± 3 ^a^	15 ± 3 ^a^ ***	20 ± 3 ^b^	18 ± 4 ^b^	19 ± 3 ^b^	14 ± 3 ^a^
			b*	14 ± 2 ^a^	17 ± 4 ^b^ **	21 ± 4 ^d^ **	19 ± 5 ^bc^	21 ± 4 ^cd^ **	17 ± 4 ^b^ **
			Δ*E*	0	8 ± 3 ^a^ **	12 ± 3 ^c^ ***	10 ± 3 ^b^	11 ± 3 ^bc^ *	7 ± 3 ^a^ **
		4 °C	L*	42 ± 1 ^d^ ***	36 ± 2 ^b^	36 ± 2 ^b^	36 ± 2 ^b^ ***	38 ± 2 ^c^ ***	35 ± 1 ^a^
			a*	17 ± 2 ^a^ *	20 ± 3 ^bc^	21 ± 3 ^c^	19 ± 4 ^ab^ **	18 ± 3 ^b^ **	22 ± 3 ^c^
			b*	15 ± 1 ^a^	18 ± 3 ^c^	19 ± 3 ^cd^	17 ± 3 ^b^ ***	4 ± 3 ^a^ ***	20 ± 3 ^d^
			Δ*E*	0	8 ± 3 ^c^	9 ± 4 ^c^	7 ± 3 ^b^ ***	5 ± 2 ^a^ **	11 ± 3 ^d^
		8 °C	L*	42 ± 1 ^c^	34 ± 2 ^b^	37 ± 2 ^a^	35 ± 2 ^a^	36 ± 2 ^b^ *	34 ± 3 ^a^ ***
			a*	16 ± 2 ^ab^	19 ± 3 ^d^	18 ± 2 ^c^	20 ± 2 ^d^	16 ± 2 ^a^ ***	17 ± 2 ^bc^
			b*	14 ± 2 ^a^	18 ± 3 ^b^	14 ± 2 ^a^	20 ± 3 ^c^	15 ± 3 ^a^ ***	18 ± 2 ^b^
			Δ*E*	0	7 ± 3 ^b^ **	5 ± 2 ^a^	10 ± 3 ^d^	6 ± 2 ^ab^ **	9 ± 2 ^c^ **

Values represent the mean ± SD of three replicates. Different letters indicate significant differences over time (*p* < 0.05). Asterisks denote treatment effects: * *p* < 0.05, ** *p* < 0.01, *** *p* < 0.001.

**Table 2:** Estimated parameters of pseudo-zero- and first-order kinetic model for quality indices of control and prototype groups of MP cherry tomatoes, stored at different temperatures up to 15 days.

Quality Indices	Reaction Order	Temperature (°C)	Groups	Kinetics Parameters		
				*k*	*R* ^2^	*RMSE*
		1	Control	0.006 ± 0.0001 ^c^	0.93	0.01
		4		0.009 ± 0.0002 ^b^	0.94	0.03
pH	Zero-order reaction	8		0.011 ± 0.0001 ^a^	0.95	0.01
		1	Prototype	0.003 ± 0.0001 ^c^ ***	0.93	0.01
		4		0.004 ± 0.0003 ^b^ ***	0.91	0.02
		8		0.009 ± 0.0002 ^a^ ***	0.96	0.01
		1	Control	0.013 ± 0.0002 ^c^	0.95	0.02
		4		0.016 ± 0.0004 ^b^	0.94	0.02
Titratable acidity	Zero-order reaction	8		0.019 ± 0.0001 ^a^	0.99	0.01
		1	Prototype	0.008 ± 0.0001 ^c^ ***	0.98	0.01
		4		0.010 ± 0.0002 ^b^ ***	0.95	0.03
		8		0.013 ± 0.0003 ^a^ ***	0.94	0.02
		1	Control	0.025 ± 0.0002 ^c^	0.82	0.06
		4		0.027 ± 0.0005 ^b^	0.83	0.05
Total polyphenol content	Zero-order reaction	8		0.031 ± 0.0010 ^a^	0.90	0.04
		1	Prototype	0.015 ± 0.0003 ^c^ ***	0.88	0.02
		4		0.019 ± 0.0010 ^b^ ***	0.91	0.06
		8		0.023 ± 0.0020 ^a^ **	0.96	0.02
		1	Control	0.064 ± 0.001 ^c^	0.95	0.05
		4		0.071 ± 0.003 ^b^	0.94	0.04
Total Antioxidant capacity	First-order reaction	8		0.083 ± 0.003 ^a^	0.94	0.06
		1	Prototype	0.031 ± 0.003 ^c^ ***	0.98	0.02
		4		0.040 ± 0.001 ^b^ ***	0.85	0.06
		8		0.051 ± 0.004 ^a^ ***	0.92	0.04

Values are reported as the mean of three replicates ± standard deviation. *k*: estimated rate reaction constants for zero and first-order model (day^−1^); *R*^2^; coefficient of determination; *RMSE*: root mean square error; Different letters show significant effect of time (*p* < 0.05); ** shows significant effect of treatment (*p* < 0.01); *** shows significant effect of treatment (*p* < 0.001).

**Table 3:** Estimated Arrhenius equation parameters for the quality indices of control and prototype groups of MP cherry tomatoes, stored at different temperatures up to 15 days.

Quality Indices	Group	*k_ref_* (day^−1^)	*E_a_* (kJ mol^−1^)	*R* ^2^
pH	Control	0.008 ± 0.01	51 ± 2 ***	0.98
	Prototype	0.005 ± 0.02	92 ± 4	0.94
Titratable acidity	Control	0.03 ± 0.02	35 ± 3 *	0.97
	Prototype	0.02 ± 0.01	46 ± 1	0.98
Total Polyphenol content	Control	0.03 ± 0.01	19 ± 3 *	0.97
	Prototype	0.02 ± 0.01	38 ± 6	0.98
Total Antioxidant capacity	Control	0.07 ± 0.02	24 ± 2 ***	0.98
	Prototype	0.04 ± 0.01	46 ± 1	0.96

*k_ref_*: estimated rate reaction constants for zero and first-order model (day^−1^) at reference temperature; *E_a_*: activation energy; *R*^2^: coefficient of determination; * shows significant effect of treatment (*p* < 0.05); *** shows significant effect of treatment (*p* < 0.001).

**Table 4:** (**a**) Microbial counts (logCFU/g) of control and prototype groups of MP cherry tomatoes, stored at 1 °C, up to 15 days. (**b**) Microbial counts (logCFU/g) of control and prototype groups of MP cherry tomatoes, stored at 4 °C, up to 15 days. (**c**) Microbial counts (logCFU/g) of control and prototype groups of MP cherry tomatoes, stored at 8 °C, up to 15 days.

Groups	Total Aerobic Microbial Count	Yeasts and Moulds	*Enterobacteriaceae*	Storage Time (Days)
(**a**)				
Prototype	3.18 ± 0.83	<1 ± 0.05	<1 ± 0.04	0
Control	3.85 ± 1.24	<1 ± 0.06	<1 ± 0.16	
Prototype	2.95 ± 0.76	<1 ± 0.31	<1 ± 0.07	2
Control	3.18 ± 1.33	<1 ± 0.08	<1 ± 0.07	
Prototype	2.38 ± 1.46	<1 ± 0.20	<1 ± 0.02	5
Control	3.28 ± 0.97	<1 ± 0.17	<1 ± 0.02	
Prototype	2.72 ± 1.10	<1 ± 0.03	<1 ± 0.20	7
Control	3.70 ± 1.58	<1 ± 0.30	<1 ± 0.15	
Prototype	2.20 ± 0.74	<1 ± 0.12	<1 ± 0.08	9
Control	3.46 ± 1.22	<1 ± 0.51	<1 ± 0.13	
Prototype	2.53 ± 1.00	<1 ± 0.09	<1 ± 0.32	15
Control	3.56 ± 1.03	<1 ± 0.11	<1 ± 0.05	
(**b**)				
Prototype	2.70 ± 1.09	<1 ± 0.08	<1 ± 0.04	0
Control	3.70 ± 1.33	<1 ± 0.10	<1 ± 0.02	
Prototype	3.48 ± 1.67	<1 ± 0.03	<1 ± 0.11	2
Control	2.89 ± 0.58	<1 ± 0.04	<1 ± 0.20	
Prototype	2.88 ± 1.10	<1 ± 0.12	<1 ± 0.07	5
Control	2.54 ± 0.56	<1 ± 0.09	<1 ± 0.09	
Prototype	<1 ± 0.09	<1 ± 0.21	<1 ± 0.07	7
Control	2.67 ± 0.91	1.65 ± 0.88	<1 ± 0.07	
Prototype	2.20 ± 1.00	<1 ± 0.04	<1 ± 0.02	9
Control	2.23 ± 1.88	<1 ± 0.17	1 ± 0.15	
Prototype	3.11 ± 0.93	3.36 ± 2.03	2.18 ± 1.23	15
Control	3.08 ± 0.96	<1 ± 0.09	2.46 ± 1.19	
(**c**)				
Prototype	2.66 ± 1.43	<1 ± 0.03	<1 ± 0.02	0
Control	1.90 ± 1.01	<1 ± 0.05	<1 ± 0.10	
Prototype	3.48 ± 1.18	1.30 ± 0.20	1 ± 0.06	2
Control	3.30 ± 1.00	<1 ± 0.03	2.20 ± 1.20	
Prototype	4.20 ± 1.89	2.45 ± 0.88	<1 ± 0.04	5
Control	4.08 ± 1.07	1.78 ± 0.47	3.38 ± 1.87	
Prototype	5 ± 1.89	3.65 ± 1.01	<1 ± 0.08	7
Control	4.36 ± 1.37	<1 ± 0.08	3.11 ± 1.34	
Prototype	4.77 ± 0.79	3.11 ± 1.10	<1 ± 0.11 ^a^	9
Control	4.72 ± 1.27	<1 ± 0.04	4.00 ± 1.35 ^b^	
Prototype	4.34 ± 0.64	3.18 ± 1.23	1.78 ± 0.25 ^a^	15
Control	4.15 ± 0.77	<1 ± 0.06	2.30 ± 0.16 ^b^	

Values are reported as the mean of three replicates ± standard deviation. Different letters show a significant effect of treatment (*p* < 0.05) between control and prototype at each sampling point.

## Data Availability

The original contributions presented in the study are included in the article, further inquiries can be directed to the corresponding author.
